# Low CD4/CD8 Ratio Is Associated with Non AIDS-Defining Cancers in Patients on Antiretroviral Therapy: ANRS CO8 (Aproco/Copilote) Prospective Cohort Study

**DOI:** 10.1371/journal.pone.0161594

**Published:** 2016-08-22

**Authors:** Mariam Noelie Hema, Tristan Ferry, Michel Dupon, Lise Cuzin, Renaud Verdon, Rodolphe Thiébaut, Camelia Protopopescu, Catherine Leport, François Raffi, Vincent Le Moing, ANRS CO 8 (APROCO/COPILOTE) study group

**Affiliations:** 1 UMI 233 IRD/Université de Montpellier, Montpellier, France; 2 Hospices civils de Lyon, Université Claude Bernard, Lyon, France; 3 CHU de Bordeaux, Bordeaux, France; 4 INSERM, UMR 1027, Toulouse, Université de Toulouse III, CHU de Toulouse, Toulouse, France; 5 CHU de Caen, EA 4655 Risques microbiens, Université de Caen, Caen, France; 6 INSERM U897, Université de Bordeaux, Bordeaux, France; 7 INSERM UMR_S 912 (SESSTIM), Université Aix-Marseille, ORS PACA, Marseille, France; 8 CMIT, 46 Rue Henri Huchard, Paris, France; 9 Infectious Diseases Department, University Hospital, Nantes, France; 10 CHU de Montpellier, Montpellier, France; University of Pittsburgh Centre for Vaccine Research, UNITED STATES

## Abstract

**Objectives:**

To study the association between CD4/CD8 ratio and morbidity in HIV-infected patients on antiretroviral therapy (ART).

**Methods:**

The APROCO/COPILOTE cohort enrolled patients initiating a protease inhibitor-containing ART in 1997–1999. The association between occurrence of first non AIDS-defining severe events (NADE) and time-dependent measures of immune restoration was assessed by 4 Cox models with different definitions of restoration, CD4+ cell counts (CD4), CD4/CD8 ratio, both CD4 and CD4/CD8 ratio, or a composite variable (CD4< 500/mm^3^, CD4 > 500/mm^3^ and CD4/CD8 ratio < 1, CD4 > 500/mm^3^ and CD4/CD8 ratio > 1). Models adjusted on baseline characteristics and time-dependent viral load were compared using Akaike Information Criterion.

**Results:**

We included 1227 patients. Median duration of follow-up was 9.2 years (IQR: 4.2–11.4). Median CD4 was 530/mm^3^ at 9 years. Median CD4/CD8 ratio was 0.3 (IQR: 0.2–0.5) at baseline and 0.6 (IQR: 0.4–0.9) after 9 years. Incidence of first NADE was 7.4/100 person-years, the most common being bacterial infections (21%), cardiovascular events (14%) and cancers (10%). For both bacterial infections and cardiovascular events, the CD4/CD8 ratio did not add predictive information to the CD4 cell count. However, low CD4/CD8 ratio was the best predictor of non-AIDS cancers (adjusted HR = 2.13 for CD4/CD8 < 0.5; 95% CI = 1.32–3.44).

**Conclusions:**

CD4/CD8 ratio remains < 1 in most HIV-infected patients despite long-term CD4+ cell counts restoration on ART. A CD4/CD8 ratio < 0.5 could identify patients who require a more intensive strategy of cancer prevention or screening.

## Introduction

Morbidity and mortality of HIV-infected people treated with combination antiretroviral therapy (cART) are now dominated by non AIDS-defining events (NADE) [1–5]. As in general population, NADE could be promoted by multiple factors, including comorbidities such as chronic hepatitis, arterial hypertension and diabetes, and high-risk behaviours such as smoking and alcohol consumption [6–7]. There is however a persistent higher incidence of morbid events in HIV-infected patients compared to the general population, which might be driven by specific factors among which immune activation linked to accelerated aging seem to be prominent [8–10].

While normalisation of CD4+ cells count above 500/mm^3^ is frequent under cART, normalisation of CD4/CD8 ratio above 1 is much slower, due mostly to persistence of elevated CD8 cell counts [11]. This persistently low CD4/CD8 ratio has been demonstrated to reflect persistent innate and adaptive immune activation in HIV-infected patients [12]. Moreover, in the general population, a low CD4/CD8 ratio is associated with risk of death in the elderly [13] and may thus be a marker of early immunosenescence in HIV-infected patients. We thus hypothesized that CD4/CD8 ratio, an easily available marker of persistent immune activation, might be a predictor of morbidity in HIV-infected patients on cART. We specifically searched to determine whether the CD4/CD8 ratio brought more information than usual immunological and virological markers in predicting the occurrence of NADE in a cohort of HIV-infected patients with a long-term follow-up on cART.

## Methods

### Patients and variables

The ANRS CO8 (Aproco/Copilote) cohort study was conducted in 47 clinical centres in France from 1997 to 2009 [14]. In total, 1281 patients were enrolled between May 1997 and June1999 at the first initiation of a protease inhibitor-containing antiretroviral therapy and followed in the cohort until December 2009. After the inclusion visit, patients were followed at 1 and 4 months after initiation of treatment, and then every 4 months. During follow-up visits, CD4+ and CD8+ cells counts, and plasma HIV RNA were updated. Severe clinical events were recorded prospectively, monitored by clinical research assistants and validated by an event validation committee [1]. All cancers were histologically proven. An event was considered severe when life threatening or leading to hospitalization or death. Non AIDS-defining severe events were those that did not fulfil the criteria for AIDS according to the 1993 CDC classification and were not obviously related to antiretroviral drugs [1]. HCV infection was defined by the presence of anti-HCV antibodies and HBV infection by the presence of HBs antigen. Socioeconomic and behavioural characteristics, including alcohol and tobacco consumptions, were collected using a self-administered questionnaire at baseline. All patients included in the APROCO/COPILOTE cohort provided written informed consent and the protocol was approved by the “Comité de Protection des Personnes se prêtant à la Recherche Biomédicale” of the Cochin Hospital (Paris).

### Statistical analysis

For the description of NADE and the analysis of potential determinants of the occurrence of the first NADE during long-term cART, we selected only NADE that occurred after the initial response to cART, i.e. after 4 months of follow-up (M4). Therefore, patients who died or were lost to follow-up before M4 were excluded from analyses.

Potential determinants of the occurrence of the first NADE after M4 were studied using Cox proportional hazards regression models. Data were censored at last follow-up in the cohort or death. Plasma HIV RNA, CD4+ cell count (CD4) and CD4/CD8 ratio recorded during follow-up after M4 as well as a composite immune restoration variable built in three categories (CD4< 500/mm^3^, CD4 > 500/mm^3^ and CD4/CD8 ratio ≤ 1, CD4 > 500/mm^3^ and CD4/CD8 ratio > 1), were treated as time-dependent variables. We first studied the association between time-dependent CD4+ cells count, CD4/CD8 ratio and the occurrence of NADE in bivariate analyses. The cut-off values for transformation of these two continuous variables into categorical variables were those having the maximal likelihood in the bivariate analyses using the Akaike information criterion (AIC). Then we constructed a multivariable model of predictors of first non-AIDS defining event, including baseline characteristics and time-dependent plasma HIV RNA level. Variables associated with a p-value of 0.25 in bivariable analysis were included in the initial multivariable model and a backward selection procedure retaining only significant variables (p < 0.05) was then used to yield a final multivariable model.

Finally, we used the AIC (the lower value being associated with maximal likelihood and thus with best prediction of outcome) to compare the four following Cox models adjusted on baseline variables and time-dependent plasma HIV RNA: (i) model with CD4, (ii) model with CD4/CD8 ratio, (iii) model with both CD4 and CD4/CD8 ratio, (iv) model with the composite immune restoration variable.

The same approach was then used to analyse the first non AIDS-defining bacterial infection, the first cardiovascular event, and the first non AIDS-defining cancer respectively. All statistical analyses were performed using SAS software, version 9.3 (SAS Institute, Inc, Cary, NC, USA).

## Results

Of the 1281 patients included in the cohort, 1227 (96%) patients having at least four months of follow-up were selected for the present analysis. The median duration of follow-up was 9.2 years, (interquartile range (IQR): 4.2–11.4). At baseline the median age was 36 years (IQR 32–43), men accounted for 77% of the sample, the main presumed mode of transmission was sex between men (40.1%) and 44.7% of patients were antiretroviral naïve. Median baseline CD4+ cells count was 278/ mm^3^ (IQR 129–422), median CD4/CD8 ratio was 0.3 (IQR 0.2–0.5) and 98.1% of patients had a CD4/CD8 ratio less than 1. Median plasma HIV RNA was 4.5 log_10_ copies/ml (IQR 3.7–5.2).

The majority of patients had immunological and virological response to cART. The proportion of patients with plasma HIV RNA < 500 copies/mL was 60% at 12 months, 87% at 9 years and 93% at 12 years. Median CD4+ cell count was 410/ mm^3^ at 12 months, 530/mm^3^ at 9 years and 585/mm^3^ at 12 years. The proportion of patients with CD4+ cells count > 500/mm^3^ was 60% at 9 years and 74% at 12 years. Median CD4/CD8 ratio increased during follow-up to reach 0.6 (IQR: 0.3–0.9) at 9 years and 0.8 (IQR: 0.5–1.1) at 12 years of follow-up, the proportion of patients having a CD4/CD8 ratio > 1 reaching 20% at 9 years and 30% at 12 years.

During follow-up, 536 of 1227 patients 43.7%) in the study had at least one NADE after M4, totalising 1152 NADE throughout follow-up (Table 1). Incidence of first NADE was 7.4/100 persons-years of follow-up. The most common were bacterial infections (21%), cardiovascular events (14%) and cancers (10%). In multivariable analysis without any measure of immune restoration, occurrence of first NADE was associated with older age, CDC stage C, HCV and HBV infection at baseline and time-dependent plasma HIV RNA level (hazard ratio = 0.70 for HIV RNA < 500 copies/mL vs > 500 copies/mL (95% confidence interval: 0.58–0.8). Associations with gender, transmission risk group, alcohol or tobacco use at baseline were not significant (Table 2).

**Table 1:** Description of the 1152 non-AIDS defining events observed in 1227 patients who had at least four months in the follow-up. APPROCO/ COPILOTE (ANRS CO8) cohort study 1997–2009.

Non-AIDS defining events	n (%)
**Bacterial infections**	**241 (20.9)**
Airway bacterial infections	122 (10.5)
Upper airway bacterial infections	7 (0.6)
Lower airway bacterial infections	115 (10.0)
Intra-abdominal infections	43 (3.7)
Skin and soft tissue infections	17 (1.5)
Anal and perianal infections	8 (0.7)
Bone and joint infections	7 (0.6)
Bacteremia	19 (1.7)
Urinary tract infections	15 (1.3)
Other bacterial infections	10 (0.9)
**Cardiovascular events**	**160 (13.9)**
**Ischemic events**	**103(8.9)**
Coronary events	59 (5.1)
Arterial events	28 (2.4)
Strokes	12 (1.0)
Other ischemic events	4 (0.4)
Non-ischemic events	26 (2.3)
Vascular events	36 (2.7)
**Cancers**	**116 (10.1)**
Solid cancers	103 (8.9)
Digestive cancers	40 (3.5)
Oesophagus	2 (0.2)
Stomach	5 (0.4)
Hepatocellular carcinoma	12 (1.0)
Pancreas	5 (0.4)
Colon and rectum	6 (0.5)
Anus	6 (0.5)
Other digestive cancer	4 (0.3)
Larynx and pharynx	14 (1.2)
Bladder and urinary tract	10 (0.9)
Lung	12 (1.0)
Skin	11 (1.0)
Other solid cancers	16 (1.4)
Hodgkin lymphoma	13 (1.1)
**Digestives events**	**97 (8.4)**
Cirrhosis	24 (2.1)
Pancreatitis	17 (1.5)
Others	56 (4.9)
**Psychiatric events**	**73 (6.3)**
Depression with or without suicide attempt	55 (4.8)
Other psychiatric disorders	18 (1.6)
**Neurological events**	**56 (4.9)**
Neurological events of central origin	34 (3.0)
Peripheral neuropathy	22 (1.9)
**Kidney and urinary tract events**	**68 (5.9)**
**Viral infections**	**34 (3.0)**
Varicella zoster infections	15 (1.3)
Other viral infections	19 (1.7)
Surgical diseases	55 (4.8)
Skin diseases	11 (1.0)
Endocrine diseases	10 (0.9)
Gynecological diseases	10 (0.9)
Hematological diseases	18 (1.6)
Events in relation with drug addiction	16 (1.4)
Ophtalmological diseases	7 (0.6)
Lung events	32 (2.8)
Rheumatological events	33 (2.9)
Trauma	40 (3.5)
General symptoms	25 (2.1)
Hypersensitivity reactions not related to cART	18 (1.6)
Deaths of unknown origin	10 (0.9)
Other events (scabies, malaria…)	22 (1.9)

**Table 2:** Factors associated with the occurrence of the first non-AIDS events. Final multivariate model without any measure of immune restoration. APPROCO/ COPILOTE (ANRS CO8) cohort study 1997–2009.

Factor studied	Non-AIDS events n (%)	Crude HR	p	Adjusted HR	95%CI	p
Age at baseline (years)			0.02			<0.001
<30	71 (38.0)	1		1		
[30–40]	247 (40.4)	0.95		0.85	0.64–1.12	
[40–50]	136 (48.6)	1.13		1.10	0.82–1.48	
[50–60]	53 (51.5)	1.25		1.34	0.93–1.93	
≥ 60	29 (63.0)	1.75		1.93	1.25–3.01	
Gender			0.63			
Male	415 (43.8)	1				
Female	121 (43.2)	1.05				
Presumed route of HIV transmission			< 0.001			
Sex between men	189 (38.4)	1				
Sex between men and women	175 (42.9)	1.19				
Intravenous drug use	105 (53.6)	1.77				
Haemophilia	9 (69.2)	2.37				
Others/unknown	40 (42.1)	1.19				
CDC stage C at baseline			0.03			0.025
No	411 (42.0)	1		1		
Yes	125 (50.4)	1.24		1.26	1.02–1.55	
Tobacco smoking at baseline			0.02			
Yes or unknown	357 (45.0)	1				
No	179 (41.3)	0.81				
Alcohol consumption at baseline			0.04			
Yes or unknown	437 (42.9)	1				
No	99 (47.6)	1.25				
HBV infection			0.04			0.021
No	504 (43.2)	1		1		
Yes	32 (54.2)	1.46		1.49	1.04–2.14	
HCV infection			< 0.001			<0.001
No	381 (40.1)	1		1		
Yes	155 (56.2)	1.64		1.76	1.45–2.15	
Time-dependent plasma HIV RNA (copies/mL)			0.001			<0.001
≥ 500	-	1		1		
< 500		0.73		0.70	0.58–0.85	

Abbreviations: HR: hazard ratio; CI: confidence interval.

When comparing adjusted models including four different measures of immune restoration during follow-up, the model associated with the maximum likelihood was the model containing only time-dependent CD4+ cells count and the higher risk of morbidity was associated with a last CD4+ cells count < 100/mm^3^, while the difference between higher strata of CD4+ cells count was minimal (Table 3).

**Table 3:** Comparison of four measures of immune restoration in prediction of occurrence of first non AIDS-defining severe events. ANRS CO8 (APROCO/COPILOTE) 1997–2009.

**Model 1 *AIC = 6772*.*54***			
**Time-dependent variable**	Adjusted* **HR**	**95% CI**	**p**
CD4+ cell count (/mm^3^)			
<100	1		
100–200	0.39	0.25–0.60	<0.001
200–500	0.43	0.30–0.61	<0.001
≥ 500	0.31	0.21–0.46	<0.001
**Model 2 *AIC = 6796*.*47***			
**Time-dependent variable**	Adjusted* **HR**	**95% CI**	**p**
CD4/CD8 ratio			
*<0*.*5*	1		
*0*.*5–1*	0.86	0.71–1.05	0.16
*≥ 1*	0.71	0.53–0.96	0.03
**Model 3 *AIC = 6787*.*38***			
**Time-dependent variable**	Adjusted* **HR**	**95% CI**	**p**
Immune restoration variable			
CD4 ≥ 500/mm^3^, CD4/CD8 ≤ 1	1		
CD4 ≥ 500/mm^3^, CD4/CD8 > 1	0.88	0.62–1.23	0.46
CD4 < 500/mm^3^	1.39	1.13–1.70	0.001
**Model 4 *AIC = 6772*.*36***			
**Time-dependent variable**	Adjusted* **HR**	**95% CI**	**p**
CD4+ cell count (/mm^3^)			
<100	1		
100–200	0.39	0.25–0.60	<0.001
200–500	0.43	0.30–0.61	<0.001
≥ 500	0.31	0.21–0.46	<0.001
CD4/CD8 ratio			
<0.5	1		
0.5–1	1.02	0.82–1.26	0.85
≥ 1	0.88	0.64–1.22	0.46

Abbreviations: AIC: Akaike Information Criterion. HR: hazard ratio; CI: confidence interval.

* Adjusted on age, stage AIDS, plasma HIV RNA level, HCV and HBV infections.

We performed the same analyses for the first non AIDS-defining severe bacterial infection, the first cardiovascular severe event, and the first non AIDS-cancer respectively. The CD4/CD8 ratio did not add supplementary prognostic information when added to the CD4+ cell count (data not shown for bacterial infections and cardiovascular events), except for the prediction of the first non-AIDS cancer for which both CD4+ cell count < 100/mm^3^ and CD4/CD8 < 0.5 were independently associated with a higher risk of event and the model containing only the CD4/CD8 ratio was associated with the higher likelihood with an HR of 2.13 for CD4/CD8 < 0.5 vs ≥ 0.5 (95% CI = 0.31–0.90) (Table 4).

**Table 4:** Comparison of four measures of immune restoration in prediction of occurrence of first non AIDS-defining cancer. ANRS CO8 (APROCO/COPILOTE) 1997–2009.

**Model 1 *AIC = 936*.*79***			
**Time-dependent variable**	Adjusted* **HR**	**95% CI**	**p**
CD4+ cell count (/mm^3^)			
<100	1		
100–200	0.22	0.05–0.92	0.04
200–500	0.35	0.13–0.92	0.03
≥ 500	0.22	0.08–0.59	0.002
**Model 2 *AIC = 932*.*16***			
**Time-dependent variable**	Adjusted* **HR**	**95% CI**	**p**
CD4/CD8 ratio			
*<0*.*5*	2.13	1.32–3.45	0.002
*≥0*.*5*	1		
**Model 3 *AIC = 938*.*41***			
**Time-dependent variable**	Adjusted* **HR**	**95% CI**	**p**
Immune restoration variable			
CD4 ≥ 500/mm^3^, CD4/CD8 ≤ 1	1		
CD4 ≥ 500/mm^3^, CD4/CD8 > 1	0.64	0.62–1.23	0.34
CD4 < 500/mm^3^	1.47	0.87–2.47	0.14
**Model 4 *AIC = 933*.*40***			
**Time-dependent variable**	Adjusted* **HR**	**95% CI**	**p**
CD4+ cell count (/mm^3^)			
<100	1		
100–200	0.23	0.05–0.96	0.04
200–500	0.43	0.16–1.14	0.09
≥ 500	0.35	0.12–1.00	0.05
CD4/CD8 ratio			
<0.5	1		
≥0.5	0.53	0.31–0.90	0.02

Abbreviations: AIC: Akaike Information Criterion. HR: hazard ratio; CI: confidence interval.

* Adjusted on age and HCV infection.

## Discussion

In the Aproco/Copilote cohort, the CD4/CD8 ratio increased progressively over time and continued to increase until 12 years of follow-up on cART, but remained below 1 for the majority of patients. The study confirmed the high incidence of NADE in this cohort and showed that these events were associated with persistence of profound immunodeficiency, i.e. very low CD4+ cell count under treatment. This association was however mainly observed in the minority of patients who had CD4 cells count remaining below 100/ mm^3^. The CD4/CD8 ratio did not add predictive information on morbidity to the CD4+ cells count, except for non-AIDS cancers for which a ratio < 0.5 constituted the main immunological predictor of these events with a twice higher risk of non AIDS cancer in patients with a CD4/CD8 ratio < 0.5.

One of the strengths of our study is the extended follow-up duration, giving both chance to observe a high number of events and time to observe immune restoration. To our knowledge, this study is the first to analyse morbidity on cART with such an extended follow-up duration. In addition, by recruiting patients in 47 medical centres in France with a standardised data collection carried out by qualified personnel, this study gave us a good representation of the population of patients who started cART in France in the late 1990s. Moreover, all severe events in the cohort were monitored, which gave us completeness in the collection of events. These events were validated by an endpoint review committee constituted by experienced clinicians (see events validation committee in appendix), which reinforces the validity of the diagnoses. These strengths probably compensate, at least in part, for the relatively small number of patients. Among the limitations of the study, the most important is probably also the recruitment of patients in the late 1990’s, during the early days of cART, where regimens were still suboptimal because of late initiation, previous exposure to nucleoside analogues and insufficient potency of the initially prescribed third agent, a first-generation unboosted protease inhibitor. The proportion of patients reaching a high CD4/CD8 ratio may be higher for patients who initiate cART nowadays with more potent combinations, although this might not be the case for late-presenters. However, because the relationship between a biological marker and morbidity is unlikely to evolve greatly over time, we believe that our main findings still apply to people currently initiating cART. Moreover, thanks to the high potency of cART, patients who initiated antiretrovirals in the 1990s still represent an important proportion of people living with HIV in industrialized countries.

In our study, despite CD4 reconstitution with cART, the CD4/CD8 ratio increased but remained below 1 for most patients. Other authors have also evidenced such a slow increase with time. Tinago et al. in Ireland, showed that only 26% of patients treated with cART had a CD4/CD8 ratio greater than 1 after 14 years of follow-up [15]. Leung et al. in Canada, studied the predictors of normalization of CD4/CD8 ratio, defined as a ratio greater than 1.2, in over 4,000 HIV-infected patients initiating cART between 2000 and 2010 with a median follow-up duration of less than 3 years. In this cohort, only 7.2% of patients had a CD4/CD8 ratio ≥ 1.2 at the end of follow-up [16]. HIV RNA suppression, high baseline CD4+ cell counts and low baseline CD8+ cell count were the main predictors of normalisation of the CD4/CD8 ratio. In the Italian cohort ICONA, the proportion of normalisation of CD4/CD8 ratio above 1 was 29.4% at 5 years of follow-up and an additional factor found associated with higher ratio was the lack of co-infection with cytomegalovirus (CMV) [17], a data that was not available in our cohort. Co-infection with CMV was also a determinant of a lower probability to reach a CD4/CD8 ratio above 1 in a transversal study conducted in Paris [18]. The mechanisms underlying the slow recovery of CD4/CD8 ratio despite the effectiveness of cART remain poorly understood. A persistent deficiency in naive CD8+ T cells [15] and/or a persistent activation of CD8+ lymphocytes linked to persistent viral production or immune activation [12] have been invoked, as well as a dysfunction of CD4+ regulatory T lymphocytes [19].

Low CD4/CD8 ratio does not seem to increase the risk of AIDS or death nowadays [16], which was not true in the earlier cART era [20]. We found that the CD4/CD8 did not provide additional information to the CD4+ cell count in predicting the now much more frequent non-AIDS-defining events, except for cancers. Our results are in discordance with those of three previous studies. Mussini et al. in a study performed in the Italian cohort ICONA found a higher risk of non-AIDS defining events in patients with a CD4/CD8 ratio < 0.3, independently of the CD4+ cell count [17]. In this study, the most frequent event was acute kidney injury, which is quite unusual. Serrano-Villar et al showed in the Madrid cohort that the CD4/CD8 ratio was significantly lower in patients suffering from non AIDS events compared to the other patients of the cohort, independently of CD4+ cells count and other prognostic factors, and this finding was consistent for all types of events including cardiovascular ones and end-stage kidney disease [21]. Of note, the number of events was low and the study used a case-control design, which is associated with a lower level of evidence than prospective studies. In the Aquitaine cohort, a study focusing on severe bacterial infections showed that these events were more frequent in patients with a CD4/CD8 ration< 0.8 [22]. A study performed in the US in patients enrolled between 1998 and 2012 showed results more in accordance with ours, i.e. a lack of association between low CD4/CD8 ratio and well-characterized NADE when adjusting for CD4+ cell counts [23]. Of note, in this latter study, when assessing NADE by category, low CD4/CD8 ratio was associated with a higher risk of coronary artery disease but not of cancer.

According to our findings, the CD4/CD8 ratio may not provide much additional information to the CD4+ cell count in predicting the occurrence of all types of non-AIDS defining events. Even if immune activation is probably a major driver of morbidity, this result is probably explained by the fact that, for most morbid events occurring in patients infected with HIV, like cardiovascular disease or neuropsychological conditions, the main drivers are probably aging, comorbidities and/or behaviour. Yet, non-AIDS-defining events are an extremely heterogeneous group that often have little in common on a pathophysiological point of view. Therefore, to study them as if they were a single event type, as is most usually done, appears questionable. In particular, the type of immune activation that favours each specific comorbidity may be at least in part specific. CD4/CD8 ratio is an indicator of CD4+ T cell lymphopenia and of CD8+ T cell activation and some comorbidities may depend on none of these factors. Our analysis showed actually that the effect of immune restoration and/or CD8+ T cell activation was quite different according to the type of event with an independent role of the CD4/CD8 ratio evidenced only on the risk of cancer. Independently of CD4+ cells count, the risk of cancer was indeed twice higher in the subset of patients who had a CD4/CD8 ratio below 0.5. A previous study, performed in patients on cART or not in the Swiss HIV cohort, is in accordance with our findings, by showing a higher risk of Hodgkin lymphoma in patients having a very low CD4/CD8 ratio < 0.25, 1 to 2 years before diagnosis of malignancy [24]. Due to the low number of events, we were not able to search for an effect of the CD4/CD8 ratio on specific types of cancers.

The CD4/CD8 ratio is easily available in clinical practice but rarely used in measuring immunological response to cART. A low CD4/CD8 ratio reflects indeed quite different situations, including low CD4+ cell count and/or persistent CD8+ T cell immune proliferation, which may preclude its clinical utility. However, our study suggests that monitoring CD4/CD8 ratio in patients receiving ART may be useful to identify a subset of patients at much higher risk of non AIDS-defining cancer who may thus require a more intensive strategy of prevention or screening.
